# Comparison of Medical Associations in Iran with Europe and America

**DOI:** 10.34172/aim.20125

**Published:** 2025-05-01

**Authors:** Nasim Hatefimoadab, Pegah Matourypour, Noredin Mohamadi, Alireza Nikbakht Nasrabadi, Hamid Peirovi, Shahrzad Ghiyasvandian, Hamid Reza Eshraghi, Mohammad Ali Cheraghi, Ali Mohammad Mosadegh Rad, Masoud Fallahi Khoshknab

**Affiliations:** ^1^Medical Surgical Nursing Department, School of Nursing and Midwifery, Kermanshah University of Medical Sciences, Kermanshah, Iran; ^2^Medical Surgical Nursing Department, Nursing and Midwifery Faculty, Tehran University of Medical Sciences, Tehran, Iran; ^3^Member of Iran Health Sciences Phenomenology Association, Nursing and Midwifery School, Tehran University of Medical Sciences, Tehran, Iran; ^4^Medical Surgical Nursing Department, Nursing and Midwifery Faculty, Iran University of Medical Sciences, Tehran, Iran; ^5^Nursing Management Department, Nursing and Midwifery Faculty, Tehran University of Medical Sciences, Tehran, Iran; ^6^Healthcare Services Management Department, Climate Change and Health Research Center (CCHRC), Health Faculty, Tehran University of Medical Sciences, Tehran, Iran; ^7^Nursing Management Department, School of Behavioral Sciences and Mental Health, University of Social Welfare and Rehabilitation Sciences, Tehran, Iran

**Keywords:** Comparative assessment, Europe, Iran, Medical association, Professional association

## Abstract

**Background:**

A comparative study of the structure, characteristics, background, and functions of scientific associations of medical sciences in Iran with other leading countries in the field of medical sciences can serve as a way forward for the future planning of these associations. This research was conducted to compare the status of medical associations in Iran and European and American countries.

**Methods:**

This is a descriptive-comparative study. After selecting scientific associations in Iran, Europe, and America, data were collected by triangulation. To validate the study’s findings, the results of the comparisons were discussed in a meeting attended by ten medical association’s representative.

**Results:**

The structure of medical associations in Iran reveals significant differences compared to their counterparts in European and American countries. The primary mission of medical associations in Iran is focused on professional development; however, their roles in policymaking are not clearly defined. All analyzed associations, whether in Iran, Europe, or the US, operate as private, non-profit entities. In terms of performance, Iranian medical associations display the highest activity levels in education (66.6%), social engagement (70%), and research (76.6%). However, they lack engagement in advisory services and advocacy-related activities. Conversely, medical associations in the US are active across all these domains, playing a significant role in healthcare.

**Conclusion:**

The results of the present study show that in Iran, most scientific associations are limited to activities in the field of education and research, while in European and American countries, they play a role in policy making and as advocates for their members.

## Introduction

 A scientific association is a specialized organization formed based on experts’ agreement and voluntary participation in a specific scientific field. These associations regulate and integrate scientific interactions and relationships between actors within scientific roles, existing in both formal and informal structures at national and international levels.^1^ Scientific associations are considered civil institutions in a society, playing a significant role in knowledge production, human resource training, and scientific development in any country.^2^ They hold a crucial position in the advancement of science and related policymaking. While scientific associations are often directly or indirectly affiliated with social institutions such as governmental universities, ministries, professional organizations, and specialized agencies, they also maintain an independent identity as part of the civil society.^3^

 The primary objectives of scientific associations include expanding and advancing knowledge and technology, fostering interdisciplinary connections, facilitating the exchange of ideas among specialists, encouraging research, strengthening research collaborations, establishing links with domestic and international scientific communities, and setting standards.^2^

 A review of the history of scientific associations in Iran indicates that before the 1960s, the scientific institutions and publications grew slowly. Among the earliest initiatives in this field were the establishment of Darul-Funun in 1846 and the Ministry of Science in 1858. Following a nearly 70-year stagnation after the cessation of Darul-Funun’s activities, the development of new scientific institutions resumed in the 1920s, culminating in establishing the University of Tehran in 1934.^4^ This phase is the first era of scientific association activities in Iran. The second era of scientific associations’ growth occurred between 1960 and 1990, during which the role of science and higher education in forming professional associations became more prominent.^5^

 Currently, the Secretariat of the Commission of Medical Scientific Associations, under the Educational Deputy of the Ministry of Health and Medical Education of Iran, is responsible for issuing licenses and renewing permits for scientific associations in the medical field.

 The American Medical Association (AMA) stands out among the leading medical associations. Established in 1847, it is the largest medical association in the United States and aims to promote and advance the art and science of medicine and improve public health. *JAMA (Journal of the American Medical Association)*, published by AMA, also holds the highest circulation among weekly medical publications worldwide.^6^

 Similarly, the European Medical Association (EMA), founded in 1990 with representatives from 12 countries, is one of the largest medical groups in Europe. EMA was established as a non-profit and independent organization to advance scientific objectives. Its member countries include Spain, Romania, Greece, Kazakhstan, Belgium, Denmark, and the United Kingdom.^7^

 The study and evaluation of the performance of medical scientific associations in Iran is a field that has largely remained unexplored by researchers. A search of reputable scientific databases suggests that this study may be the first scientific research on the structure of Iranian medical scientific associations. Since scientific associations play a key role in institutionalizing science in society, a comparative analysis of Iran’s medical scientific associations with those in Europe and the United States can provide valuable insights into their structure, processes, and performance. Identifying similarities and differences can facilitate future planning and improve the effectiveness of Iranian medical associations in the global scientific landscape. The objective of this study was to conduct a comparative analysis of the status of medical associations in Iran, Europe, and the United States.

## Materials and Methods

 This is a descriptive-comparative study. This comparative approach was carried out by initially reviewing the official website of the Medical Science Associations Commission of the Iranian Ministry of Health, Treatment, and Medical Education to extract and examine relevant data. Additionally, information from medical associations in other European and American countries was reviewed.

 For literature review and data collection, we used the keywords including “Medical association”, “Association”, “Scientific association”, and their equivalent terms in international databases such as SID, Google Scholar, PubMed, Scopus, and Web of Science.

 Furthermore, official national reports, conference proceedings, and congresses related to the research objective were reviewed to gather more information about the status of medical associations in these three countries without time restrictions.

###  Phase 1: Sampling and Selection of Medical Associations

 In the first phase of the study, a sampling process was carried out to identify medical associations in Iran, the United States, and the United Kingdom.

- The sample size included 30 associations from Iran, 30 from the United States, and 30 from the United Kingdom.
- Associations were chosen according to accreditation criteria, specifically those ranking in the top decile in at least two of seven primary categories (education and research, external relations, organizational structure, membership, management, and finance).
- A comprehensive list of associations was created based on the specified criteria. To facilitate comparisons, 65 medical associations from the United States and 81 from the U.K. which corresponded to the top-decile associations in Iran were recognized and incorporated into the research. After finalizing the list of selected scientific associations, nominal equivalence was initially used. An early review of some associations’ online platforms revealed varied but relatively acceptable similarities across the seven defined categories. Subsequently, medical associations from the United States and the U.K, which exhibited nominal equivalence to Iran’s top-decile associations, were identified and extracted.
- As a result, 30 medical scientific associations from Iran were chosen as part of the study sample. Using the same criteria, another 30 associations were selected from the 65 identified in the United States, and an additional 30 were chosen from the 81 associations found in the U.K. These associations, which corresponded to the selected Iranian scientific bodies, made up the final sample for the study.

The data collection method employed triangulation, combining multiple sources and validation techniques.

###  Phase 2: Description, Comparison, and Analysis

 The second phase of the study involved:

- Descriptive analysis: The collected data were analyzed using a checklist, developed and validated by a panel of subject matter experts. This phase resulted in a comprehensive description of the activities, objectives, mission, and vision of the selected associations.
- Comparative framework development: By juxtaposing the descriptive data, a framework was established to compare similarities and differences between associations.
- Validation of findings: The comparative analysis results were presented in a discussion group session (including 15 expert), attended by representatives from at least ten medical scientific associations. This number was chosen to ensure a diverse range of perspectives while maintaining a manageable group size for in-depth discussion. Their feedback was incorporated into the final report to refine and finalize the research outcomes.

###  Research Process Adjustments

 During the research process, two meetings were held with the research team to:

- Refine the search methodology and introduce additional relevant keywords.
- Extract historical data from collected documents, aligning with the study’s objectives.
- Utilize accreditation indicators for the final selection of scientific associations in Iran, Europe, and the United States.

The final sample selection criteria were as follows:

- Iranian medical associations: 30 associations were selected based on accreditation and categorization criteria.
- US medical associations: 30 associations were chosen from the 65 eligible medical associations.
- European medical associations (United Kingdom and other European countries): 30 associations were selected from the 81 eligible associations.

###  Qualitative Section

 Initially, a focus group discussion was conducted with experts from medical scientific associations. The session produced 102 descriptive codes, categorized under three key domains: Structure, background, and performance of medical associations.

 A contractual qualitative content analysis was applied to evaluate these categories.

 Subsequent meetings were held with the research team to:

- Define research variables,
- Develop checklist items,
- Establish methods for extracting and completing checklist data.

The face and content validity of the checklist were assessed by five subject matter experts, who provided feedback. Adjustments were made accordingly, and the final checklist was developed (Checklist 1, Supplementary file 1).

###  Ethical Considerations

- Approval for research conduct was obtained from the Ethics Committee of the Commission of Medical Scientific Associations, the National Strategic Medical Education Research Center, and the Educational Deputy of the Ministry of Health, Treatment, and Medical Education.
- Research findings were shared with the relevant units and officials upon request.
- Ethical publishing principles (COPE) were adhered to, ensuring proper citation of sources when translating materials into Persian.
- Integrity and fairness were maintained in sampling, data collection, and analysis.
- Confidentiality of information was strictly observed.

## Results

 Based on the qualitative data analysis conducted in this study, the findings are categorized into three main domains:

1. Structure
2. Context
3. Performance

The key findings regarding the structure of medical scientific associations are as follows:

- Membership in scientific associations is voluntary. If membership is mandatory, the entity is not classified as an association but a governmental regulatory body, similar to the Iranian Medical Council.
- Organizations such as the AMA oversee all scientific associations while maintaining close ties with the legislative body and playing an active role in governance.
- The structure of medical associations includes various components, such as:
  o Mission and objectives
  o Formation process (by consensus or election)
  o Membership policies
  o Existence of an active digital system (website, platform, or registry)
  o Communication mechanisms with regulatory bodies
  o Stakeholder engagement processes
  o Accountability measures
  o Public or private nature of the association
  o Licensing authority for issuing permits or certificates
  o Organizational structure and governing bodies
  o Committees and subcommittees

The key findings regarding the context of medical scientific associations include:

- Laws and regulations governing associations
- Processes for establishment and formation
- Licensing and operational permit procedures
- Funding sources and financial resource mobilization strategies
- Taxation policies or eligibility for tax exemption
- Governmental or judicial oversight and monitoring mechanisms
- Authority and degree of operational independence

The key findings concerning the performance of medical scientific associations in the medical sciences domain include:

- The primary mission of these associations is to advocate for the rights of the medical community.
- The most significant function of scientific associations is advisory.
- Depending on each country’s socio-political and governance structure, different professional groups may be designated as associations, councils, or organizations.
- Membership in these associations is free, and membership fees are covered by taxation policies defined in legal frameworks.
- In some countries, each association’s board of directors, councils, and committees are responsible for governance functions, with these responsibilities formally assigned by constitutional law.

Further details regarding the mission of medical scientific associations include:

- Promoting social participation
- Facilitating education and training programs
- Conducting problem-oriented research
- Providing mechanisms for policy implementation
- Delivering professional services to members
- Establishing and improving educational and research standards for public benefit
- Publishing academic and general-interest materials for both the public and association members.

The results of the checklist-based assessment related to structure, context, and performance are presented in Tables 1 to 3.

**Table 1:** Comparison of the Structure of Medical Associations by Country

**Structure Items**	**Countries**					
	**The UK (** ***n*** **=30)**		**The US (** ***n*** **=30)**		**Iran (** ***n*** **=30)**	
	**Percent**	**Number**	**Percent**	**Number**	**Percent**	**Number**
Defined mission (professional, policymaking)	70.0	21	83.3	25	86.6	26
Membership fee requirement	63.3	19	60	18	56.6	17
Membership benefits for members	60.0	18	63.3	19	26.6	8
Having an updated website	60.0	18	56.6	17	40.0	12
Communication with regulatory bodies	50.0	15	53.3	16	63.3	19
Having a license or permit to operate	36.6	11	43.3	13	30.0	9
Non-profit nature of the association	100	30	100	30	100	30
Timely holding of annual general meetings/elections	26.6	8	43.3	13	40.0	12
Defined composition of specialized committees	43.3	13	46.6	14	36.6	11
Defined composition of the board of directors/members	66.6	20	56.6	17	96.6	29

**Table 2:** Comparison of the Context of Medical Associations by Country.

**Context Items**	**Countries**					
	**The UK (n=30)**		**The US (n=30)**		**Iran (n=30)**	
	**Percent**	**Number**	**Percent**	**Number**	**Percent**	**Number**
History and formation process of the association	43.3	13	43.3	13	80.0	24
Tax exemption history	—	—	13.3	4	—	—
Defined rules and regulations	20.0	6	20	6	—	—
Financial resource mobilization process	—	—	3.33	1	6.6	2
Association performance oversight	—	—	—	—	—	—
Defined scope of authority and autonomy	6.6	2	—	—	—	—
Documented services provided to members	16.6	5	26.6	8	—	—
Acceptance of the association among graduates of the field	—	—	—	—	—	—
Accreditation (external evaluation)	—	—	—	—	—	—

**Table 3:** Comparison of the Performance of Medical Associations by Country

**Performance Items**	**Countries**					
	**The UK (n=30)**		**The US (n=30)**		**Iran (n=30)**	
	**Percent**	**Number**	**Percent**	**Number**	**Percent**	**Number**
Education	63.3	19	56.6	17	66.6	20
Educational calendar						
Educational curriculum						
Educational catalog or podcast						
Professional competency exams						
Educational guidelines						
Organizing training courses/organizing conferences						
Clinical services	43.3	13	50	15	20.0	6
Having a virtual network of clinical/field managers						
Clinical management training						
Patient safety and quality of care						
Center accreditation reports						
Patient/family education						
Counseling services	3.3	15	6.6	2	—	—
Research	50.0	15	56.6	17	76.6	23
Providing grants and research budgets						
Calendar of research events						
Organizing working groups and training and research courses						
Having a magazine						
Advocacy	13.3	4	30	9	—	—
Social participation	56.6	17	50	15	70.0	21
Public publications/services						

## Discussion

 The structure of medical associations in Iran reveals significant differences compared to European and American countries. The primary mission of medical associations in Iran is focused on professional development; meanwhile, their roles in policymaking are not clearly defined. In contrast, medical associations in the U.K and the United States actively engage in health policymaking and governance. Laugesen and Gusmano highlighted that physician associations in high-income countries play a vital role in shaping health policies, while their influence in low-income countries, like Iran, is notably limited. This discrepancy may be closely tied to the varying stages of health system development across these countries.^8^ Furthermore, Glauser emphasized that providing diverse benefits and services to members is essential for increasing membership engagement.^9^ Unfortunately, many Iranian scientific associations neglect this aspect, often failing to outline membership benefits on their websites.

 Huynh and Chung argue for the necessity of a well-defined organizational structure within medical associations, including a clearly stated mission and a defined membership base. They assert that successful associations, such as the AMA, integrate factors such as mutual trust, constructive interaction, commitment, accountability, collective success, and strong leadership into their frameworks. In Iran, although 63.3% of medical associations explicitly state their relationship with regulatory bodies like the Ministry of Health, this relationship is primarily advisory. In contrast, US medical associations maintain clear definitions of their interactions with regulatory agencies, ensuring transparency in governance.^10^

 All analyzed associations, whether in Iran, Europe, or the US, operate as private, non-profit entities. However, the only context-related information accessible for Iranian scientific associations pertains to historical data and establishment processes for 24 associations (80%). Other critical elements, such as performance oversight, acceptance among field graduates, and accreditation, remain largely unaddressed on their official websites. This lack of transparency indicates a need for Iranian medical associations to focus more on contextual factors to enhance their development.

**Figure 1:**
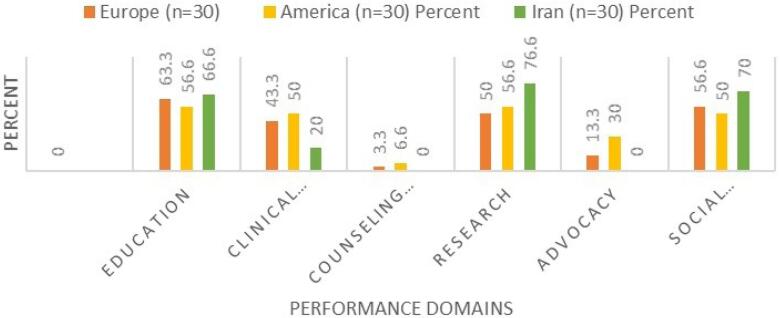
Comparison of the Performance of Medical Associations by Country

 In terms of performance, Iranian medical associations display the highest activity levels in education (66.6%), social engagement (70%), and research (76.6%). However, they lack engagement in advisory services and advocacy-related activities, with the lowest levels of activity observed in service provision (20%), international engagement (20%), and the development of formal educational and research standards (16.6%). Conversely, medical associations in the US are active across all these domains, playing a significant role in healthcare (Figure 1).

 While Iranian medical associations are highly active in education and research—similar to their US and UK counterparts—the range of educational services offered is limited. In Iran, educational activities primarily consist of workshops, webinars, and informational pamphlets, whereas US associations provide comprehensive curricula, educational catalogs, professional development guidelines, credentialed training programs, and mobile applications. Research activities in Iran are mostly confined to publishing journals and organizing conferences, lacking the availability of research grants and funding that is common in the US and UK. This disparity in resources and opportunities can hinder the advancement of medical knowledge and innovation within Iran, ultimately affecting patient care and healthcare outcomes.

 Regarding clinical practice, Iranian medical associations do not have a significant presence, unlike their US and UK counterparts, which actively influence clinical affairs. Rothman indicated that professional medical associations are vital for improving the quality of healthcare by means of education, establishing practice standards, and engaging in advocacy efforts.^11^ Additionally, Indar et alemphasized the significance of these associations in transforming health systems by providing support to their members and partnering with various stakeholders.^12^

 In summary, medical associations have the potential to play multifaceted roles in health systems, including shaping health policy,^13^ strengthening public health,^14^ advocating for disease prevention,^15^ and addressing health equity.^16^ The evolving role of medical associate professionals in the UK. healthcare system underscores the importance of collaboration in healthcare delivery, enhancing service efficiency and patient outcomes.^17^ Iranian medical associations must take a more active role in these matters to enhance their influence on the healthcare system. This change could include better collaboration with policymakers, building partnerships with universities, and supporting research projects that tackle health issues specific to the region.

## Conclusion

 The examination of the structure and functions of medical associations in Iran reveals critical gaps when compared to their counterparts in high-income countries such as the United States and European nations. Although the primary emphasis of Iranian medical associations is on enhancing professional skills, their limited involvement in health policy formulation and advisory capacities significantly undermines their possible influence on the healthcare system. Lack of transparency regarding membership benefits and the absence of a comprehensive organizational framework further hinder their growth and effectiveness.

## Recommendation

 To improve the influence and efficacy of medical associations in Iran, it is recommended that medical associations:

1. Enhance their involvement in the policy process
2. Attempt to promote health equity and public health programs
3. Strive to redefine their structure and context-related activity.

## Supplementary Files

Supplementary file 1. Inspection checklist.
