# Correction: Comparison of Medical Associations in Iran with Europe and America

**DOI:** 10.34172/aim.35101

**Published:** 2025-08-01

**Authors:** Nasim Hatefimoadab, Pegah Matourypour, Noredin Mohamadi, Alireza Nikbakht Nasrabadi, Hamid Peirovi, Shahrzad Ghiyasvandian, Hamid Reza Eshraghi, Mohammad Ali Cheraghi, Ali Mohammad Mosadegh Rad, Masoud Fallahi Khoshknab

**Affiliations:** ^1^Medical Surgical Nursing Department, School of Nursing and Midwifery, Kermanshah University of Medical Sciences, Kermanshah, Iran; ^2^Medical Surgical Nursing Department, Nursing and Midwifery Faculty, Tehran University of Medical Sciences, Tehran, Iran; ^3^Member of Iran Health Sciences Phenomenology Association, Nursing and Midwifery School, Tehran University of Medical Sciences, Tehran, Iran; ^4^Medical Surgical Nursing Department, Nursing and Midwifery Faculty, Iran University of Medical Sciences, Tehran, Iran; ^5^Nursing Management Department, Nursing and Midwifery Faculty, Tehran University of Medical Sciences, Tehran, Iran; ^6^Healthcare Services Management Department, Climate Change and Health Research Center (CCHRC), Health Faculty, Tehran University of Medical Sciences, Tehran, Iran; ^7^Nursing Management Department, School of Behavioral Sciences and Mental Health, University of Social Welfare and Rehabilitation Sciences, Tehran, Iran

 In the article titled "Comparison of Medical Associations in Iran with Europe and America," published in *Archives of Iranian Medicine* (2025;28(5):296-302; doi: 10.34172/aim.20125), the School name in Affiliation number 1 was not formatted correctly. The correct affiliation format is as follows: Medical Surgical Nursing Department, School of Nursing and Midwifery, Kermanshah University of Medical Sciences, Kermanshah, Iran.

 This correction has been updated in both the PDF and HTML versions of the article.

