# Supplementary material for: Comparison of Medical Associations in Iran with Europe and America
Source: Arch Iran Med. 2025 May 1;28(5):296–302. doi: 10.34172/aim.20125 (PMC12305405; doi:10.34172/aim.20125)
Supplement: Supplementary file 1 — Inspection checklist. [file aim-28-296-s001.pdf]

Arch Iran Med. May 2025;28(5):296-S1
10.34172/aim.20125
http://journalaim.com

Supplementary file 1

Checklist 1. Inspection checklist.

NO	Yes	Item	
***Defined mission as:**			Structure
		Professional	
		Policymaking	
***Membership**			
		Benefits for members	
		Requirement for membership	
		Requirement for payment of membership fee	
***Active website**			
		Having a license or permit to operate	
		The associations website update status	
		Communication with regulatory bodies	
***Association members**			
		Timely holding of annual general meetings (ordinary and extraordinary)	
		Timely holding of board of directors elections	
		Number of members approved	
		Defined composition of the board of trustees	
		Defined composition of specialized committees	
		Defined composition of members	
		History and formation process of the association	Context
		Tax exemption history	
		Defined rules and regulations	
		Financial resource mobilization process	
		Association performance oversight	
		Defined scope of authority and autonomy	
		Documented services provided to members	
		Acceptance of the association among graduates of the field	
***Role**			Performance
		Education	
		Clinical service	
		Consulting services	
		Research	
		Advocacy	
		Social participation	
